# The ocrelizumab wearing-off phenomenon is associated with reduced immunomodulatory response and increased neuroaxonal damage in multiple sclerosis

**DOI:** 10.1007/s00415-024-12434-w

**Published:** 2024-05-23

**Authors:** Isabel Monteiro, Valerio Nicolella, Mariano Fiorenza, Federica Novarella, Antonio Carotenuto, Roberta Lanzillo, Lucia Mauriello, Giulia Scalia, Giuseppe Castaldo, Daniela Terracciano, Vincenzo Brescia Morra, Marcello Moccia

**Affiliations:** 1 Department of Molecular Medicine and Medical Biotechnology, Federico II University of Naples, Naples, Italy; 2 Multiple Sclerosis Unit, Policlinico Federico II University Hospital, Via Sergio Pansini 5, 80131 Naples, Italy; 3 Neurology Department, Coimbra University Hospital Center, Coimbra, Portugal; 4 Department of Neuroscience, Reproductive Sciences and Odontostomatology, Federico II University of Naples, Naples, Italy; 5 Department of Translational Medical Science, Federico II University of Naples, Naples, Italy; 6 Centre for Advanced Biotechnology (CEINGE), Naples, Italy

**Keywords:** Multiple sclerosis, Ocrelizumab, Wearing-off, Lymphocyte, Neurofilament

## Abstract

**Objective:**

The wearing-off phenomenon is common in people with multiple sclerosis (MS) treated with ocrelizumab. We aim to evaluate the presence and severity of wearing-off to ocrelizumab in relation to demographic and MS clinical variables, immune profiling, and a marker of neuroaxonal damage (plasma neurofilament light chain (pNfl)).

**Methods:**

This cross-sectional study included MS patients treated with ocrelizumab from at least 1 year. Wearing-off questionnaire and blood samples were collected between 21 and 23 weeks after the previous ocrelizumab infusion. Lymphocyte subpopulations were evaluated on peripheral blood using flow cytometry. PNfl was evaluated using fully automated chemiluminescent enzyme immunoassay.

**Results:**

We included 106 people with MS (age 49.5 ± 11.6 years; females 42.3%; wearing-off 57.6%). On regression models, wearing-off was associated with higher pNfl, CD8, CD3, and CD3CD27 lymphocytes. Most frequent wearing-off symptoms were cognitive, sensory, and balance problems; wearing-off started < 1 week (9.4%), 1–4 weeks (10.7%) or > 4 weeks (10.7%) before infusion; 44.8% of the complaints were moderate to severe. Severity of wearing-off was associated with higher pNfl and CD8 lymphocytes.

**Conclusions:**

Wearing-off is common in people with MS treated with ocrelizumab, and is associated with reduced immunomodulation (higher T lymphocytes) and increased neuroaxonal damage, suggesting reduced treatment response.

## Background

Ocrelizumab is a humanized anti-CD20 monoclonal antibody that is approved for the treatment of relapsing and primary progressive multiple sclerosis (MS), and acts primarily through the depletion of B cells [1]. Ocrelizumab is administrated intravenously every 6 months, though many patients report on wearing-off symptoms before the end of 6-month dosing interval [2]. The “wearing-off phenomenon” was initially described in people with MS treated with natalizumab, and consists of increased MS-related symptoms (i.e., fatigue, cognitive disability, balance problems, motor dysfunction, and sensory symptoms), which disappear immediately or in the next days following the infusion. While wearing-off affects about 50% of people with MS treated with ocrelizumab, its mechanisms are largely unexplored [2, 3]. In one study [3], younger age and higher disability were associated with the presence of wearing-off. In another study [2], authors found body mass index (BMI) to be correlated with this phenomenon. Nonetheless, in both studies, no associations were found between wearing-off and serum neurofilament light chain (NfL), a marker of neuroaxonal damage, thus implying the subjective nature of this phenomenon. However, wearing-off might reflect subclinical response to treatment, that could be measured using immune profiling [4]. Also, a number of confounders, including age, cardiovascular comorbidities [5–7], and BMI [8], might affect treatment response and related markers (e.g., Nfl), and were not previously accounted for.

As such, we aim to evaluate: (1) demographic and clinical correlates of the wearing-off phenomenon in a population of MS patients treated with ocrelizumab; (2) differences in immune profiling in relation to presence and severity of the wearing-off phenomenon; (3) differences in Nfl in relation to presence and severity of the wearing-off phenomenon.

## Methods

### Study design and population

This is a cross-sectional study conducted at the Multiple Sclerosis Clinical Unit of the Federico II University Hospital, Naples, Italy. The study was approved by the Federico II Ethics Committee (332/21). All patients signed informed consent authorizing the use of anonymized data in line with data protection regulation (GDPR EU2016/679). The present study was performed in accordance with good clinical practice and Declaration of Helsinki.

Inclusion criteria were: (1) diagnosis of MS according to 2017 revision of McDonald Criteria [9]; (2) age > 18 years; (3) treatment with ocrelizumab from at least 1 year (corresponding to at least 2 infusions of 600 mg).

Exclusion criteria were: (1) cognitive impairment or other conditions limiting the ability to fill in the questionnaires, as determined by clinical neurologist; (2) history of severe traumatic brain injury or stroke; (3) treatment with alemtuzumab or B-cell-depleting therapies other than ocrelizumab in the 12 months before starting on ocrelizumab; (4) use of steroids or other treatments potentially affecting lymphocytes; (5) significant alcohol or substance misuse; (6) impaired renal function; (7) hypoalbuminemia; (8) type 2 diabetes.

Patients were asked to participate in the study at their scheduled neurological consultation and blood drawn, between 21 and 23 weeks after the previous ocrelizumab infusion.

### Wearing-off questionnaire

The wearing-off questionnaire was previously developed and validated [2, 3], and, for this study, was translated in Italian (preliminary iterations were evaluated for the ease of comprehension by five patients). The wearing-off questionnaire contains five questions. In particular, participants were asked: (1) if they ever experienced wearing-off phenomenon (never, sometimes, usually, always); (2) if they felt the need for infusion at the current visit (yes, no, unsure); (3) what were their wearing-off symptoms (fatigue, cognitive disability, sensory symptoms, balance problems, walking difficulties, coordination dysfunction, muscle weakness, bladder problems, pain, or not answered); (4) how many weeks before the ocrelizumab infusion these symptoms usually appear (< 1 week; 1–4 weeks; > 4 weeks; or not answered); and (5) the severity of their wearing-off related complaints (mild, moderate, severe, or not answered). For statistical purposes, from question 3, we calculated the total number of wearing-off symptoms.

### Demographics and clinical variables

We included the following variables: age, sex, height, weight (from which we calculated the BMI), smoking (ever or never smoker), cardiovascular comorbidities (high blood pressure, high cholesterol, diabetes, atrial fibrillation, stroke, coronary disease and/or related medications), MS disease duration (time from reported onset to assessment), expanded disability status scale (EDSS) score, descriptor of disease course (relapsing, progressive), previous disease-modifying treatment (DMT), interval dosing regimen (standard vs extended), total duration of ocrelizumab infusions, and total number of ocrelizumab infusions. For statistical purposes, we classified previous DMTs in none (treatment naïve patients), platform (dimethyl fumarate, glatiramer acetate, interferon, teriflunomide), and high efficacy (cladribine, fingolimod and other S1P modulators, natalizumab, rituximab).

### Immune profiling

Fasting blood samples were obtained on the same day of the clinical assessments. An aliquot (50 μL) of anti-coagulated ethylene-diamine-tetra-acetic acid (EDTA) whole fresh blood (within 12 h) was incubated at 4 °C for 30 min in the presence of appropriate amounts of monoclonal antibodies. The mixtures were then diluted 1:20 in ammonium chloride lysing solution, incubated at room temperature for 10 min, and finally washed prior to flow cytometric analysis with the FACSCanto II flow cytometer (Becton Dickinson, San Jose, CA, USA). Samples were analyzed on FACSDiva software (BD Bioscience, San Jose, CA, USA). The following antigens were analyzed: CD4 PE (from BD San Diego, CA, USA), CD8 APCcy7 (from Beckman Coulter, Marseille Cedex 9, France), CD20 FITC (from BD San Diego, CA, USA), CD19 APC (from Beckman Coulter, Marseille Cedex 9, France), CD3 Pacific Blu (from Beckman Coulter, Marseille Cedex 9, France), CD56 PEcy7 (from Beckman Coulter, Marseille Cedex 9, France), and CD27 FITC (from BD San Diego, CA, USA). Lymphocytes were gated on forward scatter (FSC) and side scatter (SSC) parameters, identifying 50,000 events. The lowest level of detection was 10^–4^ (as such, zero corresponds to a level below 1/10,000 cells). For lymphocyte absolute count, we coupled cytometry to complete blood count on hematological counter (double platform). Laboratory procedures were performed in accordance with UK-NEQAS quality standards (https://ukneqas.org.uk/).

### Neurofilament light chain

Fasting blood samples were collected on the same day of the clinical assessments. Plasma was obtained by centrifugation (1100 rpm × 10 min) of whole blood within 3 h from collection, and, then, aliquoted and stored into polypropylene tubes at -80 °C, according to the manufacturer’s recommendations. Plasma neurofilament light chain (pNfL) levels were evaluated using fully automated chemiluminescent enzyme immunoassay (LUMIPULSE®, Fujirebio, Tokyo, Japan), consisting of two-step immunoassay method on the Lumipulse G system. The result are expressed in picograms per milliliter (pg/mL).

### Statistical analyses

Mean (and standard deviation) (age, BMI, MS disease duration, total duration of ocrelizumab infusions, and total number of ocrelizumab infusions), median (and range) (EDSS), and number (and percent) (sex, smoking, presence of cardiovascular comorbidities, descriptor of disease progression, and previous DMTs) were calculated for different study variables.

To achieve aim 1 (demographic and clinical correlates of the wearing-off phenomenon), we used different univariable linear (age, BMI, MS disease duration, EDSS, total duration of ocrelizumab infusions, total number of ocrelizumab infusions) and multinomial logistic (sex, smoking, presence of cardiovascular comorbidities, descriptor of disease progression, previous DMT) regression models, including each demographic and clinical variable, in turn, as dependent variable, and question 1 of the wearing-off questionnaire, as independent variable (if the wearing-off phenomenon was experienced never, sometimes, usually, or always). Then, the same models were run including the full set of covariates (age, sex, BMI, MS disease duration, EDSS, descriptor of disease progression, previous DMT, total duration of ocrelizumab infusions, total number of ocrelizumab infusions, smoking, and presence of cardiovascular comorbidities).

To achieve aim 2 and 3 (differences in immune profiling and pNfl in relation to presence and severity of the wearing-off phenomenon), we used different univariable linear regression models including each laboratory variable (lymphocyte subpopulations and pNfl), in turn, as dependent variable, and question 1 of the wearing-off questionnaire, as independent variable (if the wearing-off phenomenon was experienced never, sometimes, usually, or always). Then, the same models were run including the full set of covariates (age, sex, BMI, MS disease duration, EDSS, descriptor of disease progression, previous DMT, total duration of ocrelizumab infusions, total number of ocrelizumab infusions, smoking, and presence of cardiovascular comorbidities). If the presence of wearing-off phenomenon was established in both unadjusted and adjusted models, the severity was then evaluated using univariable linear regression models including each laboratory variable (lymphocyte subpopulations and pNfl), in turn, as dependent variable, and each additional question of the wearing-off questionnaire (2, 3, 4, and 5), in turn, as independent variable. Then, the same models were run including the full set of covariates (age, sex, BMI, MS disease duration, EDSS, descriptor of disease progression, previous DMT, total duration of ocrelizumab infusions, total number of ocrelizumab infusions, smoking, and presence of cardiovascular comorbidities).

Results were reported as coefficients (Coeff), odds ratio (OR), 95% confidence interval (95%CI), and *p* values, as appropriate. Distribution of variables and residuals was checked using both graphical and statistical methods. Statistical analyses were performed using Stata 15.0 (StataCorp, College Station, TX, USA). Results were considered statistically significant if *p* < 0.05.

## Results

We included 106 people with MS (age 49.5 ± 11.6 years; sex 42.3% females), with 12.7 ± 7.6 years of disease duration and median EDSS score of 4.5 (from 1.0 to 7.5). Looking at previous treatments, 25.5% of the patients were naïve to treatment, while the remaining received previous DMTs. All patients received a standard-interval dose (< 6 months’ interval from previous infusion); the mean number of ocrelizumab infusions was 6.4 ± 3.4. The mean pNfl was 17.1 pg/ml, ranging from 3.7 to 106.3 pg/ml. Full demographic, clinical, and treatment features are reported in Table 1. No patient had clinical or radiological activity at the time of the assessment.

**Table 1:** Demographic, clinical, and treatment features

	*N* = 106
Age, years	49.5 ± 11.6
Sex, females (%)	66 (62.2%)
Disease duration, years	12.7 ± 7.6
EDSS, median (range)	4.5 (1.0–7.5)
Disease descriptor	
Relapsing	46 (43.4%)
Progressive	60 (56.6%)
Previous DMT	
Naive	27 (25.5%)
Platform treatment	90 (84.9%)
High efficacy treatment	78 (73.6%)
Number of previous DMTs	
0	27 (25.5%)
1	31 (29.2%)
2	25 (23.6%)
> 3	23 (21.7%)
Ocrelizumab treatment	
Duration of treatment, months	32.4 ± 20.0
Number of infusions	6.4 ± 3.4
Cardiovascular risk factors	28 (26.4%)
Ever smoking	27 (25.4%)
BMI	25.4 ± 4.0
Plasma NfL, pg/ml	17.1 ± 16.8

Out of 106 patients, 55 patients (51.8%) reported on wearing-off before ocrelizumab infusion and 35 (33.0%) mentioned the need for infusion at the current visit. Cognitive disability (10.4%), sensory symptoms (10.4%), balance problems (10.4%) and walking difficulties (7.5%) were the most commonly-reported symptoms. The beginning of the wearing-off was < 1 week before infusion in 8.5% patients, 1–4 weeks before infusion in 24.5%, and more than 4 weeks before infusion in 24.5%. Wearing-off symptoms were moderate or severe in 39.6% of the patients (Table 2).

**Table 2:** Wearing-off questionnaire

	*N* = 106
WO1—ever experienced wearing-off phenomenon	
Never	45 (42.5%)
Sometimes	34 (32.1%)
Usually	16 (15.1%)
Always	11 (10.4%)
WO2—need for infusion at the current visit	
Yes	35 (33.0%)
No	44 (41.5%)
Unsure	27 (25.5%)
WO3—reported symptoms	
Fatigue	6 (5.6%)
Cognitive disability	11 (10.4%)
Sensory symptoms	11 (10.4%)
Balance problems	11 (10.4%)
Walking difficulties	8 (7.5%)
Coordination dysfunction	4 (3.8%)
Muscle weakness	5 (4.7%)
Bladder problems	4 (3.8%)
Pain	1 (0.9%)
Not answered	45 (42.5%)
WO4—start wearing-off before infusion	
< 1 week before infusion	9 (8.5%)
1–4 weeks before infusion	26 (24.5%)
> 4 weeks before infusion	26 (24.5%)
Not answered	45 (42.5%)
WO5—severity of complaints	
Mild	19 (17.9%)
Moderate	34 (32.1%)
Severe	8 (7.5%)
Not answered	45 (42.5%)

We found no significant associations between presence of wearing-off phenomenon and demographic and clinical variables in the adjusted model (Table 3).

**Table 3:** Demographic and clinical correlates of the wearing-off phenomenon

Unadjusted models					Adjusted models			
		95% CI				95% CI		
(No wearing-off as reference)		Lower	Upper	*p* values		Lower	Upper	*p* values
Age								
Sometimes	Coeff − 6.03	− 11.11	− 0.95	**0.02**	Coeff − 3.96	− 8.70	0.78	0.10
Usually	Coeff 2.66	− 3.84	9.17	0.42	Coeff 0.82	− 4.81	6.46	0.77
Always	Coeff − 0.13	− 7.63	7.40	0.98	Coeff − 3.13	− 9.84	3.59	0.36
Sex								
Sometimes	OR − 0.60	− 1.53	0.32	0.20	OR − 0.74	− 1.83	0.35	0.18
Usually	OR − 0.96	− 2.23	0.30	0.14	OR − 1.07	− 2.41	0.27	0.12
Always	OR − 0.42	− 1.78	0.93	0.54	OR − 0.88	− 2.44	0.69	0.27
MS duration								
Sometimes	Coeff − 4.57	− 7.92	− 1.22	**<0.01**	Coeff − 2.80	− 5.84	0.24	0.07
Usually	Coeff − 0.98	− 5.27	3.31	0.65	Coeff − 2.55	− 6.14	1.03	0.16
Always	Coeff − 0.81	− 5.76	4.15	0.75	Coeff − 3.66	− 7.92	0.61	0.09
EDSS								
Sometimes	Coeff − 0.10	− 0.87	0.67	0.80	Coeff 0.51	− 0.12	1.15	0.11
Usually	Coeff 0.76	− 0.22	1.76	0.13	Coeff 0.62	− 0.13	1.37	0.10
Always	Coeff 1.08	− 0.05	2.23	0.06	Coeff 0.83	− 0.06	1.72	0.07
Disease descriptor								
Sometimes	OR 0.25	− 0.64	1.14	0.58	OR − 0.12	− 1.52	1.27	0.86
Usually	OR − 0.65	− 1.86	0.55	0.29	OR − 0.29	− 1.90	1.32	0.72
Always	OR − 1.37	− 3.01	0.27	0.10	OR − 1.08	− 3.33	1.17	0.35
Duration ocrelizumab								
Sometimes	Coeff 5.03	− 4.05	14.14	0.28	Coeff 0.39	− 0.39	1.18	0.32
Usually	Coeff 0.96	− 10.68	12.62	0.87	Coeff − 0.10	− 1.02	0.82	0.83
Always	Coeff 3.27	− 10.18	16.74	0.63	Coeff 1.03	− 0.05	2.11	0.06
Number of ocrelizumab infusions								
Sometimes	Coeff 0.81	− 0.71	2.34	0.29	Coeff − 0.06	− 0.19	0.07	0.36
Usually	Coeff 0.18	− 1.77	2.14	0.85	Coeff 0.02	− 0.14	0.17	0.83
Always	Coeff 0.38	− 1.87	2.65	0.73	Coeff − 0.17	− 0.35	0.01	0.06
Previous DMTs								
Sometimes	OR 1.29	0.27	2.32	**0.01**	OR 0.46	− 0.95	1.88	0.52
Usually	OR 0.06	− 1.40	1.54	0.93	OR − 0.58	− 2.5	1.33	0.55
Always	OR − 0.77	− 2.96	1.42	0.49	OR − 1.26	− 4.06	1.53	0.38
BMI								
Sometimes	Coeff 0.46	− 1.40	2.34	0.62	Coeff 0.78	− 1.34	2.90	0.47
Usually	Coeff 0.44	− 1.88	2.78	0.71	Coeff 0.72	− 1.77	3.20	0.57
Always	Coeff 1.54	− 1.14	4.24	0.26	Coeff 1.67	− 1.29	4.63	0.27
Smoking								
Sometimes	OR 0.39	− 0.59	1.38	0.44	OR 0.75	− 0.41	1.9	0.21
Usually	OR − 0.81	− 2.44	0.81	0.33	OR − 0.72	− 2.42	0.99	0.41
Always	OR 0.14	− 1.34	1.64	0.85	OR 0.44	− 1.21	2.08	0.6
CV risk factors								
Sometimes	OR − 1.06	− 2.19	0.07	0.66	OR − 1.17	− 2.65	0.3	0.12
Usually	OR − 0.09	− 1.32	1.13	0.88	OR − 0.35	− 1.76	1.07	0.63
Always	OR − 0.28	− 1.75	1.18	0.70	OR − 1.06	− 2.91	0.78	0.26

Table shows coefficients (Coeff), odds ratio (OR), 95% confidence intervals (95% CI), and p values from univariable linear (age, BMI, MS disease duration, EDSS, total duration of ocrelizumab infusions, total number of ocrelizumab infusions) and multinomial logistic (sex, smoking, presence of cardiovascular comorbidities, descriptor of disease progression, previous DMT) regression models, including each demographic and clinical variable, in turn, as dependent variable, and question 1 of the wearing-off questionnaire, as independent variable (if the wearing-off phenomenon was experienced never, sometimes, usually, or always). Then, the same models were run including the full set of covariates (age, sex, BMI, MS disease duration, EDSS, descriptor of disease progression, previous DMT, total duration of ocrelizumab infusions, total number of ocrelizumab infusions, smoking, and presence of cardiovascular comorbidities). Significant results (*p* < 0.05) are reported in bold

**Fig. 1:**
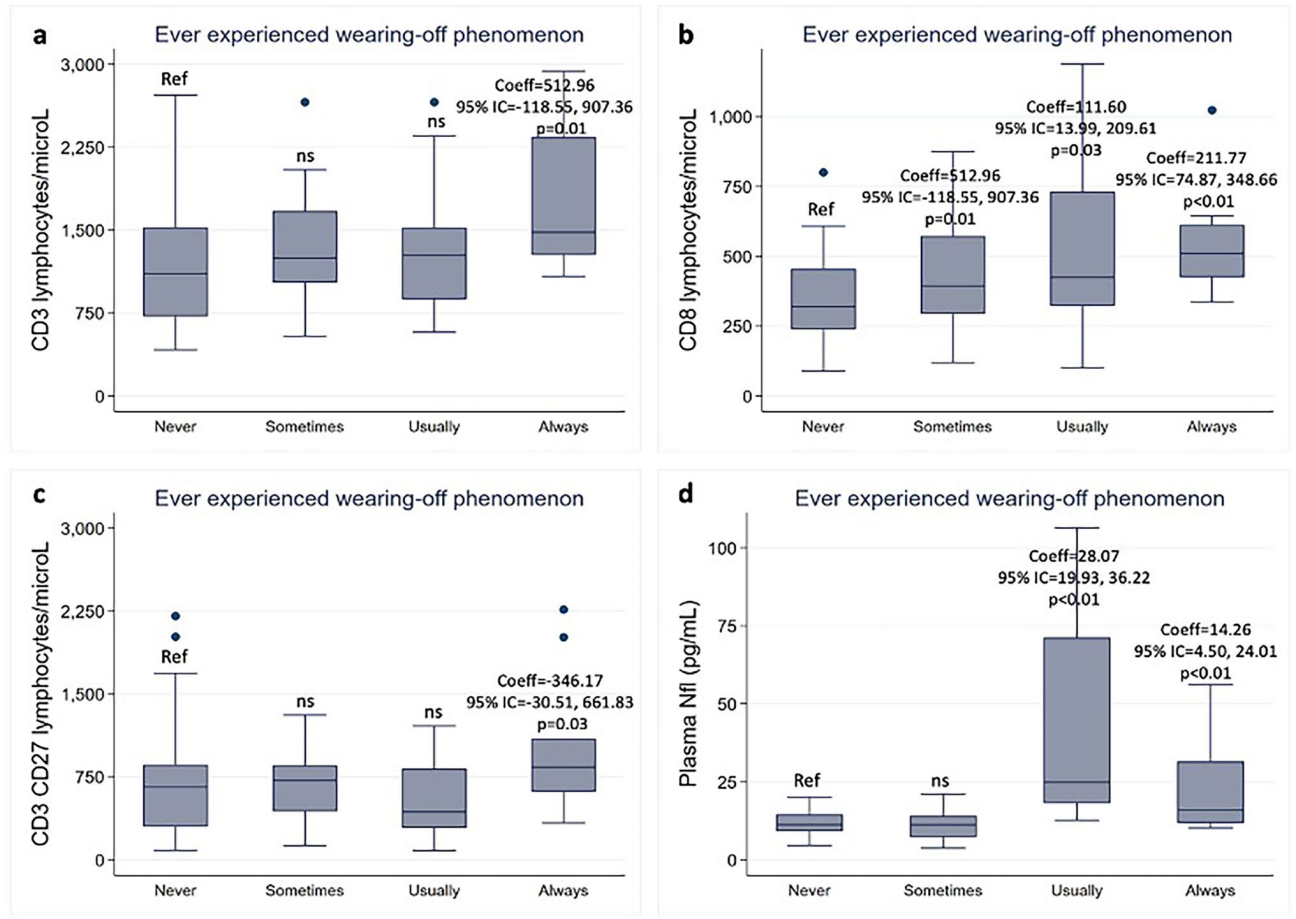
Laboratory correlates of the presence of the wearing-off phenomenon. Box plots show the associations between the wearing-off phenomenon and levels of CD3 lymphocytes (a), CD8 lymphocytes (b), CD3CD27 lymphocytes (c); and plasma neurofilaments light chain (Nfl) (d). Coefficients (Coeff), 95% confidence intervals (95% CI), and p values are represented for significant associations. Ref – Reference; ns – non significant

**Fig. 2:**
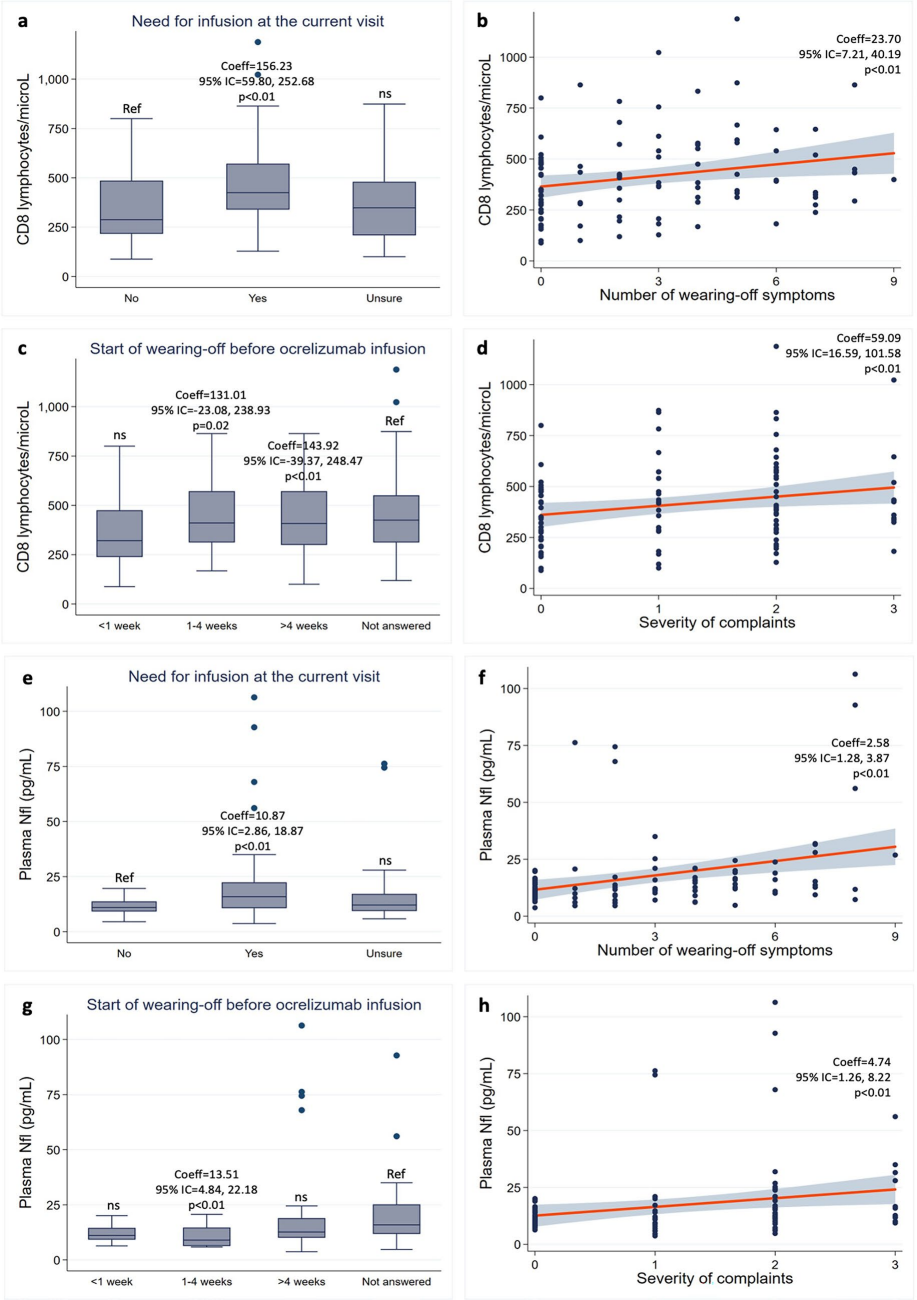
Laboratory correlates of the severity of the wearing-off phenomenon. Box plots show the associations between the need for infusion at the current visit and CD8 lymphocytes (a) and plasma Nfl (e), and between the start of wearing-off before infusion and CD8 lymphocytes (c) and plasma Nfl (g). Scatter plots (grey shades represent confidence intervals) show the associations between the number of reported symptoms and CD8 lymphocytes (b) and plasma Nfl (f), and between the severity of complaints and CD8 lymphocytes levels (d) and plasma Nfl (h). Coefficients (Coeff), 95% confidence intervals (95% CI), and p values are represented for significant associations. Ref – Reference; ns – non significant

Looking at the immune profile, patients with wearing-off had higher levels of CD8, CD3 and CD3CD27 lymphocytes, but no significant differences in CD19, CD20, CD56, CD4, CD27, and CD19CD27 lymphocytes (Table 4). In particular, patients experiencing wearing-off “always” had higher CD8 (Coeff = 211.76; 95% CI = 74.86, 348.66; *p* < 0.01), CD3 (Coeff = 512.95; 95% CI = 118.55, 907.36; *p* = 0.01), and CD3CD27 lymphocytes (Coeff = 346.17; 95% CI = 30.50, 661.83; *p* = 0.03). Patients experiencing wearing-off “usually” had higher CD8 lymphocytes (Coeff = 218.16; 95% CI = 103.78, 332.53; *p* < 0.01). Patients with the need for ocrelizumab infusion at the current visit had higher CD8 (Coeff = 156.23; 95% CI = 59.79, 252.68, 348.66; *p* < 0.01) and CD3 lymphocytes (Coeff = 275.83; 95% CI = 7.93, 543.73; *p* = 0.04). Patients with more severe wearing-off had higher levels of CD8 lymphocytes, but no significant differences in CD3 and CD3CD27 lymphocytes. In particular, there were higher CD8 lymphocytes in patients with more severe symptoms (Coeff = 59.09; 95% CI = 16.59, 101.58; *p* < 0.01), with emergence of symptoms 1–4 weeks (Coeff = 131.01; 95% CI = 23.08, 238.93; *p* = 0.02) and more than 4 weeks before infusion (Coeff = 143.92; 95% CI = 39.37, 248.47; *p* < 0.01), and with more wearing-off symptoms (Coeff = 23.70; 95% CI = 7.21, 40.19; *p* < 0.01) (Table 4). Of note, levels of CD19 and CD20 lymphocytes were approximately zero (0.6 ± 2.3/μL and 0.5 ± 2.3/μL, respectively) (Figs. 1,2).

**Table 4:** Differences in lymphocytes in relation to the wearing-off phenomenon

Unadjusted models					Adjusted models			
		95% CI				95% CI		
	Coeff	Lower	Upper	*p* Values	Coeff	Lower	Upper	*p* Values
CD3								
WO1 (never as reference)								
Sometimes	91.04	− 159.99	342.07	0.47	52.55	228.65	333.74	0.71
Usually	128.61	− 192.94	450.16	0.43	172.78	− 156.74	502.29	0.30
Always	504.70	133.12	876.26	**<0.01**	512.96	118.55	907.36	**0.01**
WO2 (no as reference)								
Yes	262.48	10.38	514.57	**0.04**	275.83	7.93	543.73	**0.04**
Unsure	224.40	− 60.70	509.49	0.12	326.35	23.03	629.67	**0.04**
WO3 (number of symptoms)	27.81	− 13.45	69.06	0.18	28.22	− 18.15	74.59	0.23
WO4 (no as reference)								
<1 week	179.79	− 224.11	583.69	0.38	114.09	− 355.35	583.52	0.63
1–4 weeks	118.07	− 162.15	398.28	0.41	96.74	− 210.66	404.15	0.53
>4 weeks	117.31	− 162.91	397.52	0.41	124.43	− 173.37	422.23	0.41
WO5 (need for infusion)	62.17	− 43.39	167.74	0.25	75.42	− 43.53	194.36	0.21
CD4								
WO1 (never as reference)								
Sometimes	− 4.34	− 181.99	173.33	0.96	− 59.89	− 262.35	142.57	0.56
Usually	− 65.80	− 293.38	161.77	0.57	− 54.89	− 292.14	182.36	0.65
Always	294.22	31.25	557.20	**0.03**	284.37	0.39	568.34	0.05
CD8								
WO1 (never as reference)								
Sometimes	91.30	4.35	178.25	0.04	111.60	13.99	209.21	0.03
Usually	182.345	70.97	293.72	**<0.01**	218.16	103.79	332.53	**<0.01**
Always	196.07	67.38	324.78	**<0.01**	211.77	74.87	348.66	**<0.01**
WO2 (no as reference)								
Yes	131.09	41.88	220.30	**<0.01**	156.23	59.8	252.68	**<0.01**
Unsure	56.12	− 44.77	157.01	0.27	86.50	− 22.71	195.70	0.12
WO3 (number of symptoms)	18.18	3.61	32.76	**0.02**	23.70	7.21	40.19	**<0.01**
WO4 (no as reference)								
<1 week	108.76	− 32.74	250.26	0.13	151.64	− 13.17	316.44	0.07
1− 4 weeks	99.12	0.96	197.29	**0.05**	131.01	23.08	238.93	**0.02**
>4 weeks	118.43	20.27	216.60	**0.02**	143.92	39.37	248.47	**<0.01**
WO5 (need for infusion)	44.73	7.33	82.13	**0.02**	59.09	16.59	101.58	**<0.01**
CD4/CD8								
WO1 (never as reference)								
Sometimes	− 0.43	− 0.96	0.10	**0.01**	− 0.59	− 1.22	0.03	0.06
Usually	− 0.78	− 1.46	− 0.10	**0.03**	− 0.91	− 1.65	− 0.18	**0.02**
Always	0.33	− 1.11	0.46	0.41	− 0.32	− 1.2	0.55	0.47
WO2 (no as reference)								
Yes	− 0.32	− 0.84	0.2	0.23	− 0.43	− 1.02	0.16	0.15
Unsure	0.45	− 0.14	1.04	0.14	0.47	− 0.21	1.14	0.17
WO3 (number of symptoms)	− 0.77	− 0.16	0.01	0.08	− 0.1	− 0.20	0.01	0.05
WO4 (no as reference)								
<1 week	− 0.70	− 1.53	0.14	0.10	− 1.09	− 2.10	− 0.73	0.36
1–4 weeks	− 0.30	− 0.88	0.28	0.31	− 0.50	− 1.17	0.16	0.14
>4 weeks	− 0.56	− 1.13	0.02	0.06	− 0.67	− 1.31	− 0.02	**0.04**
WO5 (need for infusion)	− 0.20	− 0.42	0.02	0.07	− 0.24	− 0.51	0.02	0.07
CD19								
WO1 (never as reference)								
Sometimes	7.92	− 6.97	22.81	0.29	7.34	− 7.86	22.55	0.34
Usually	− 3.5	− 22.57	15.58	0.72	− 1.84	− 19.66	15.98	0.84
Always	− 7.02	− 29.06	15.02	0.53	− 8.61	− 29.94	12.72	0.43
CD20								
WO1 (never as reference)								
Sometimes	7.92	− 6.97	22.81	0.29	7.34	− 7.86	22.55	0.34
Usually	− 3.50	− 22.57	15.58	0.72	− 1.84	− 19.66	15.98	0.84
Always	− 7.02	− 29.06	15.02	0.53	− 8.61	− 29.72	12.72	0.43
CD56								
WO1 (never as reference)								
Sometimes	− 102.80	− 180.12	− 25.48	**0.01**	− 76.73	− 163.55	10.09	0.08
Usually	10.76	− 86.39	107.92	0.83	8.61	− 91.57	108.80	0.87
Always	− 63.81	− 180.40	52.77	0.28	− 59.24	− 183.44	64.95	0.35
CD27								
WO1 (never as reference)								
Sometimes	− 45.39	− 268.68	177.89	0.69	− 57.57	− 306.21	191.07	0.65
Usually	− 161.16	− 447.18	124.85	0.27	− 77.06	− 368.60	214.47	0.60
Always	318.31	− 25.23	661.83	0.07	346.20	− 9.94	702.34	0.06
CD3CD27								
WO1 (never as reference)								
Sometimes	3.83	− 191.58	199.23	0.97	− 26.35	− 246.73	194.03	0.81
Usually	− 128.49	− 378.79	121.81	0.31	− 67.59	− 325.99	190.81	0.60
Always	329.77	29.14	630.40	**0.03**	346.17	30.51	661.83	**0.03**
WO2 (no as reference)								
Yes	118.60	− 80.27	317.47	0.24	138.37	− 71.95	348.69	0.19
Unsure	178.62	− 45.13	402.38	0.12	275.72	38.23	513.21	**0.02**
WO3 (number of symptoms)	− 4.81	− 37.20	27.58	0.77	− 0.60	− 37.13	35.20	0.97
WO4 (no as reference)								
<1 week	4.89	− 320.77	310.98	0.98	− 39.54	− 406.91	327.83	0.83
1–4 weeks	− 40.51	− 259.66	178.63	0.72	− 73.670	− 314.32	166.98	0.54
>4 weeks	− 39.38	− 260.73	181.97	0.73	− 3.8	− 237.3	229.69	0.97
WO5 (need for infusion)	− 15.05	− 98.79	68.69	0.72	4.94	− 88.79	98.67	0.92
CD19CD27								
WO1 (never as reference)								
Sometimes	− 3.14	12.11	5.84	0.49	− 2.93	− 12.84	6.99	0.56
Usually	− 4.56	− 16.05	6.94	0.43	− 3.81	− 15.44	7.82	0.52
Always	− 5.08	− 18.89	8.73	0.47	− 5.17	− 19.36	9.03	0.47

Table shows coefficients (Coeff), 95% confidence intervals (95% CI), and *p* values from univariable linear regression models including each laboratory variable (lymphocyte subpopulations), in turn, as dependent variable, and question 1 of the wearing-off questionnaire, as independent variable (if the wearing-off phenomenon was experienced never, sometimes, usually, or always). Then, the same models were run including the full set of covariates (age, sex, BMI, MS disease duration, EDSS, descriptor of disease progression, previous DMT, total duration of ocrelizumab infusions, total number of ocrelizumab infusions, smoking, and presence of cardiovascular comorbidities). If the association with wearing-off was established in both unadjusted and adjusted models, the severity was then evaluated using univariable linear regression models including each laboratory variable (lymphocyte subpopulations), in turn, as dependent variable, and each additional question of the wearing-off questionnaire (2, 3, 4, and 5), in turn, as independent variable. Then, the same models were run including the full set of covariates (age, sex, BMI, MS disease duration, EDSS, descriptor of disease progression, previous DMT, total duration of ocrelizumab infusions, total number of ocrelizumab infusions, smoking, and presence of cardiovascular comorbidities). Significant results (*p* < 0.05) are reported in bold

Looking at pNfl, patients experiencing wearing-off “always” (Coeff = 14.25; 95% CI = 4.50, 24.01; *p* < 0.01) and “usually” (Coeff = 28.07; 95% CI = 19.93, 36.22; *p* < 0.01) had higher pNfl. Similarly, higher pNfl was found in patients with the need for ocrelizumab infusion at the current visit (Coeff = 10.87; 95% CI = 2.86, 18.87; *p* < 0.01), with more severe symptoms (Coeff = 4.74; 95% CI = 1.26, 8.22; *p* < 0.01), with emergence of symptoms 1–4 weeks before infusion (Coeff = 13.511; 95% CI = 4.84, 22.18; *p* < 0.01), and with more wearing-off symptoms (Coeff = 2.58; 95% CI = 1.28, 3.87; *p* < 0.01) (Table 5).

**Table 5:** Differences in pNfl in relation to the wearing-off phenomenon

Unadjusted models					Adjusted models			
		95% CI				95% CI		
	Coeff	Lower	Upper	*p* Values	Coeff	Lower	Upper	*p* Values
WO1 (never as reference)								
Sometimes	− 0.51	− 6.54	5.52	0.87	1.70	− 5.26	8.65	0.63
Usually	28.50	20.78	36.23	**<0.01**	28.07	19.93	36.22	**<0.01**
Always	10.97	2.04	19.90	**0.02**	14.26	4.50	24.01	**<0.01**
WO2 (no as reference)								
Yes	10.15	2.80	17.51	<0.01	10.87	2.86	18.87	**<0.01**
Unsure	6.30	− 2.02	14.62	0.14	5.65	− 3.41	14.72	0.22
WO3 (number of symptoms)	2.1	0.94	3.26	**<0.01**	2.58	1.28	3.87	**<0.01**
WO4 (no as reference)								
<1 week	− 1.15	− 12.63	10.33	0.84	0.15	− 13.09	13.4	0.98
1–4 weeks	0.06	2.090	18.02	**0.01**	13.51	4.84	22.18	**<0.01**
>4 weeks	9.17	1.21	17.14	**0.02**	10.71	2.31	19.11	0.13
WO5 (need for infusion)	3.83	0.77	6.9	2.48	4.74	1.26	8.22	**<0.01**

Table shows coefficients (Coeff), 95% confidence intervals (95% CI), and *p* values from univariable linear regression models including pNfl as dependent variable, and question 1 of the wearing-off questionnaire, as independent variable (if the wearing-off phenomenon was experienced never, sometimes, usually, or always). Then, the same models were run including the full set of covariates (age, sex, BMI, MS disease duration, EDSS, descriptor of disease progression, previous DMT, total duration of ocrelizumab infusions, total number of ocrelizumab infusions, smoking, and presence of cardiovascular comorbidities). If the association with wearing-off was established in both unadjusted and adjusted models, the severity was then evaluated using univariable linear regression models including pNfl as dependent variable, and each additional question of the wearing-off questionnaire (2, 3, 4, and 5), in turn, as independent variable. Then, the same models were run including the full set of covariates (age, sex, BMI, MS disease duration, EDSS, descriptor of disease progression, previous DMT, total duration of ocrelizumab infusions, total number of ocrelizumab infusions, smoking, and presence of cardiovascular comorbidities). Significant results (*p* < 0.05) are reported in bold

## Discussion

Our study confirmed the presence of wearing-off phenomenon in the majority of people with MS treated with ocrelizumab, irrespective of demographic, clinical and treatment features. Also, we showed that the presence of wearing-off was associated with laboratory measures pointing toward reduced modulation of T lymphocytes (e.g., higher levels of CD8 and CD3CD27 lymphocytes) and increased neuroaxonal damage (e.g., pNfl).

In our study, 57.6% people with MS treated with ocrelizumab had wearing-off phenomenon (sometimes in 32.1%, usually in 15.1%, and always in 10.4%), which is in line with two previous studies [2, 3]. Notably, fatigue was not the most common symptom, as for natalizumab wearing-off [10], but we found that cognitive, sensory, and balance problems were the most prevalent complaints, possibly as a consequence of the high representation of progressive forms of MS in our study sample (56.6% progressive MS vs 43.4% relapsing MS), that frequently have these symptoms [9].

Looking at demographic, clinical, and treatment features, we did not find any differences between the groups with and without wearing-off. On the contrary, Killestein and colleagues [2] found an association between higher BMI and wearing-off phenomenon, as in natalizumab studies [11]. Indeed, patients with higher BMI might have lower ocrelizumab concentrations and lower modulation of lymphocytes [8], which, in turn, was associated with disability progression [12, 13]. Also, Kister and colleagues [3] found an association between the presence of wearing-off and lower ocrelizumab concentrations before infusion in a univariate logistic regression. Overall, these studies preliminary suggested that the wearing-off phenomenon could be a consequence of reduced treatment response, as from higher distribution volume (e.g., BMI) and lower therapeutic concentrations.

The nature of wearing-off phenomenon is unknown, though likely related to inflammatory aspects of MS. Looking at natalizumab wearing-off studies, conflicting results also exist. While some authors propose a pharmacokinetic explanation [10], others support a placebo effect, not identifying any relation between natalizumab concentration or alpha4 integrin receptors saturation with the experience of wearing-off [14]. Considering ocrelizumab mechanism of action (anti-CD20 B lymphocytes monoclonal antibody), first, we focused on CD19 and CD20 B lymphocytes, but failed to find any significant association, as in Killestein and colleagues [2], with very low levels, not supporting evidence of B-cell reconstitution. Indeed, B lymphocytes are the main target of ocrelizumab therapy, decreasing immediately after the first infusion, and then remaining stable if infusions are done regularly (as in our population). However, we decided to evaluate T lymphocytes as well, which are also modulated by ocrelizumab and progressively decrease over infusions. In particular, we found that the presence and severity of wearing-off was associated with higher CD8 lymphocyte counts, as from reduced modulation. Previous studies showed that higher counts of CD8 T cytotoxic lymphocytes were associated with an increased probability of disability progression, suggesting their contribution to chronic inflammation within the central nervous system [4, 15, 16]. We also found higher counts of CD3 + CD27 + lymphocytes, which are involved in the generation and long-term maintenance of T-cell immunity [8]. As such, the increase in symptoms toward the end of the ocrelizumab infusion cycle might correspond to reduced treatment response, as reflected by reduced modulation of T lymphocytes and subsequent risk of disability progression [4].

In keep with this, we found higher pNfl in relation to presence and severity of wearing-off. Blood-based measurements of Nfl have been gaining relevance within the management of MS, as a marker of neuroaxonal damage [6]. In MS, Nfl has been associated with inflammatory activity [7], progression [17], prognosis [18, 19], and treatment response [17, 20]. In a re-analysis of phase 3 clinical trials, ocrelizumab demonstrated to immediately decrease NfL, following its activity on relapses and disability progression [21]. However, authors identified a subgroup of patients with residual increase of Nfl that was associated with long-term progression [21]. In our study, we showed that higher levels of pNfl during ocrelizumab treatment were associated with presence and severity of wearing-off symptoms, thus suggesting this is a clinical marker of subclinical response and risk of progression. Of note, our patients did not present with clinical or MRI activity at the time of the study, thus suggesting our pNfl values correspond to non-relapsing progressive pathobiology of MS. Interestingly, Killestein and colleagues [2] and Kister and colleagues [3] failed to found any association between Nfl and wearing-off phenomenon. However, their studies included mostly relapsing patients with lower Nfl levels, when compared to our study with a majority of progressive patients, with subsequent statistical power implications [22]. Also, they did not fully account for concomitant factors affecting Nfl levels, including BMI, smoking, and cardiovascular comorbidities [23]. From a technical standpoint, they measured serum Nfl using Simoa technology, whereas we assessed plasma Nfl by LUMIPULSE immunoassay; while the two methods have very high agreement [24], results remain not fully comparable.

Considering the large amount of studies evaluating extended interval dosing of ocrelizumab [25–28], our results raise concerns over its feasibility and long-term efficacy. If we are to assume that the wearing-off phenomenon is a marker of suboptimal treatment response, we envisage a more individualized approach in clinical practice. In particular, the high prevalence of the wearing-off phenomenon (about 50% of treated patients across different studies) suggests caution in extending the dosing intervals to everyone. Patients without wearing-off might indeed be eligible for extended interval dosing, likely preventing hypogammaglobulinemia and/or infections without compromising sustained treatment efficacy [29]. On the contrary, patients experiencing wearing-off should stay within regulatory intervals for ocrelizumab infusions or, in the future, might be considered for higher doses of ocrelizumab, pending efficacy and safety results in ongoing clinical trials (GAVOTTE; NCT0454899) (MUSETTE; NCT04544436). Not least, general considerations on the wearing-off phenomenon in people with MS treated with ocrelizumab might be then translated to a broader population within a framework of poor treatment response and disease progression [9].

Limitations of our study include the use of a questionnaire specifically designed for the wearing-off phenomenon, that, in turn, might increase the awareness of participants with overestimation of symptoms. In the future, wearing-off symptoms and their changes should be addressed within a validated tool accounting for different patient-reported outcome measures. Second, we conducted a cross-sectional study, that is unable to draw any causal relationship, and longitudinal evaluation is warranted, also to obtain objective data on actual progression. Finally, assessing ocrelizumab concentration would provide more accurate information regarding pharmacokinetics and related laboratory changes [30].

In conclusion, we confirmed that the wearing-off phenomenon is a common complaint of people with MS treated with ocrelizumab, independently from demographic, clinical, and treatment features. Presence and severity of wearing-off were associated with reduced immunomodulation of T lymphocytes and increased neuroaxonal loss, that could lead to worse clinical outcomes in the long term.

## Data Availability

Data is available upon reasonable request to the corresponding author.
